# The Impact of Lactobacillus Plantarum PCS26 Supplementation on the Treatment and Recurrence of Urinary Tract Infections in Children—A Pilot Study

**DOI:** 10.3390/jcm11237008

**Published:** 2022-11-27

**Authors:** Katarina Meštrović Popovič, Petra Povalej Bržan, Tomaž Langerholc, Nataša Marčun Varda

**Affiliations:** 1 General Hospital Celje, Oblakova 5 (omit Splošna bolnišnica Celje), SI-3000 Celje, Slovenia; 2 Faculty of Medicine, University of Maribor, Taborska ulica 8, SI-2000 Maribor, Slovenia; 3 Faculty of Electrical Engineering and Computer Science, University of Maribor, Koroška cesta 46, SI-2000 Maribor, Slovenia; 4 Department of Microbiology, Biochemistry, Molecular Biology and Biotechnology, Faculty of Agriculture and Life Sciences, University of Maribor, Pivola 10, SI-2311 Hoče, Slovenia; 5 Department of Pediatrics, University Medical Centre Maribor, Ljubljanska cesta 2, SI-2000 Maribor, Slovenia

**Keywords:** *Lactobacillus plantarum*, probiotics, prophylaxis, urinary tract infections (UTI), uropathogenic *E. coli* (UPEC)

## Abstract

Urinary tract infections (UTI) are frequent bacterial infections in childhood. Considering the known beneficial effects of probiotics in the gastrointestinal field, they could also help to alleviate UTIs. In our clinical pilot study, we sought to verify the positive effects of the specific probiotic strain on the course and prevention of UTI in children. Thirty children with UTIs were enrolled and sequentially sampled into two groups (placebo/control and probiotic/test) in a double-blind, randomized, placebo-controlled clinical pilot study. We chose *Lactobacillus plantarum* PCS 26 (Lp26) derived from local Slovenian cheese in Pathogen Combat Project, which showed a good in vitro antimicrobial effect on *Escherichia coli (E. coli).* Several parameters were followed to look for differences between both groups in the acute phase of the UTI and after 6 months of taking probiotic or placebo supplementation. Our results showed no statistically significant differences between both groups; however, two children in the placebo group suffered a recurrence of febrile UTI within 6 months of the follow-up period, while there were no recurrences of UTI in the probiotic group. In the test group, the number of febrile days after the initiation of antibiotics with probiotics was shorter, although not reaching statistical significance (*p* = 0.084). According to our results, probiotics might be helpful in alleviating UTI symptoms and in UTI prevention. Further research with a larger sample size is warranted. Additionally, basic scientific studies for the selection of proper immunobiotic strains of probiotics should be performed.

## 1. Introduction

Urinary tract infections (UTI) are a highly common pathology in childhood, with significant morbidity and hospitalization rates. The majority of UTIs are the result of colonic bacteria ascending from the periurethral region to the bladder and further to the upper urinary tract. Clinical manifestations of UTI vary with age and localization [1,2] and significant bacteriuria with ≥10^5^ CFU/mL (colony forming units per milliliter) is necessary for the diagnosis of UTIs [3]. In pathogenesis of acute UTI and even in long-term effects on renal parenchyma, innate immunity plays a crucial role [4]. The host’s innate immune system usually prevents the excessive growth of bacteria in the urinary tract using different mechanisms, among them also chemokines and cytokines. When a UTI is triggered, a complex cascade of events including upregulation of pro-inflammatory cytokines is initiated. Some authors suggest that IL-6 might be a useful biomarker for UTI detection, due to the significant correlation between intensity of the UTI and concentration of IL-6 in urine and serum [4,5,6]. IL-8 is the most important chemokine and activator of neutrophils [4]. During the UTI it attracts neutrophils to cross the mucosa and is elevated in the urine, which has been shown in adults and children [5].

In some children, UTIs recur [6,7,8,9,10]. The most important part of approaching these children is early recognition and treatment of the UTI [11], as well as some preventive measures (e.g., antibiotic prophylaxis) [12]. However, in recent years problems with resistant bacteria have emerged [13]. Therefore, new methods for the prevention of UTIs have been suggested, including the use of probiotics [14,15].

Probiotics are living microorganisms that can, when administered in adequate amounts, alter the microbiota mix and confer a health benefit on the host with potential immunomodulatory and metabolic effects as well as the normalization of intestinal microbiota composition [16]. These effects have been described in numerous studies of gastrointestinal infections, allergic diseases, systemic chronic diseases, and urogenital infections in adult women [17,18,19,20,21,22]. Studies investigating the role of probiotics in UTIs in children are scarce [12]. Studies of probiotics in managing UTIs confirmed their positive effect when added to antibiotic prophylaxis; however, so far it was not confirmed that probiotics themselves significantly lower the incidence and recurrence rate of UTIs [22]. It has been shown that specific probiotics affect conditions and diseases differently [21]. The same is true for combined preparations, therefore in vitro testing of probiotics, which can aid in the selection of strains proven to be effective in their action against uropathogens, has been developed [20]. It has been suggested that probiotics may positively alter the gut environment, downregulate the mucosal secretory response to pathogens, and activate a local immune response [17].

In our clinical pilot study, we sought to verify the positive effects of the specific probiotic strain on the course and prevention of UTIs in children according to the mechanism of probiotic function in the gastrointestinal tract and lower urinary tract [18,21]. The probiotics act as competitors to pathogen bacteria in the gut, thereby reducing important pathogens that cause UTI, such as *E. coli*, which might lower the risk or even prevent the UTI due to the decreased concentration of pathogens in the gut and also in the stool. We chose *Lactobacillus plantarum PCS 26 (Lp26)* derived from local Slovenian cheese in the “Pathogen Combat Project” which showed a good in vitro antimicrobial effect on *E. coli* in the project [16]. Lp26 as a dietary supplement led to some beneficent clinical and laboratory effects in patients with metabolic syndrome [16]. We believe that it might be of benefit also in other uropathogens.

The aim of our study was to assess the possible beneficial effects of *Lp26* on UTIs in children. We expected a milder course of UTI, less frequent recurrences of UTI, and a more beneficial influence on the child’s general health. With a milder and shorter duration of UTI, we could significantly reduce the discomfort suffered by children and their parents in dealing with and overcoming the acute infection and the need for hospitalization.

## 2. Materials and Methods

**Figure 1:**
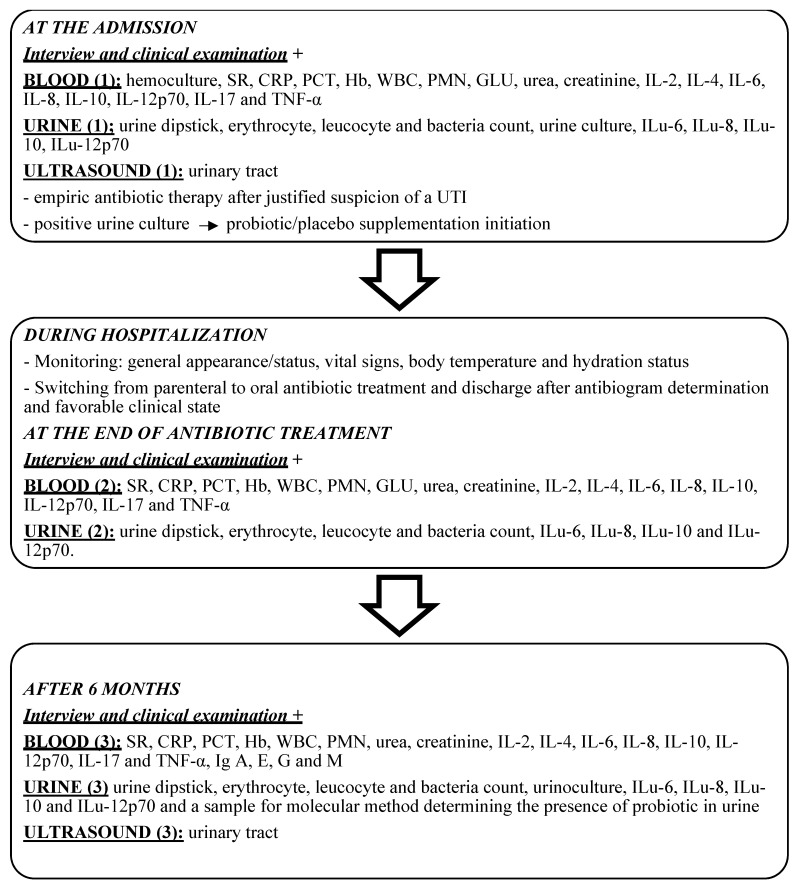
Flowchart of present study design and workflow. SR—sedimentation rate, CRP—C-reactive protein, PCT—procalcitonin, Hb—haemoglobin, WBC—white blood cell count, PMC—% polimorphonuclear cells, IL-2—interleukin-2, IL-4—interleukin-4, IL-6—interleukin-6, IL-8—interleukin-8, IL-10—interleukin-10, IL-12p70—interleukin-12p70, IL-17—interleukin-17, TNFα—tumor necrosis factor alpha, Ilu-6—urinary interleukin-6, Ilu-8—urinary interleukin-8, Ilu10—urinary interleukin-10, Ilu12p70—urinary interleukin-12p70, US—ultrasound, IgA, E, G, M—inmmunoglobulin A, E, G, M.

Thirty children with febrile UTI admitted to General Hospital Celje in 2016 were enrolled and sequentially sampled into two groups (red and yellow), in a double-blind study, all with the same protocol. The study design and workflow are presented in the flowchart (Figure 1).

The study was approved by the National Ethical Committee of Slovenia (approval code 101/09/09) and the written consent of all parents or legal guardians was obtained.

The inclusion criteria were as follows:

- age above 2 months,
- first UTI (infection of any part of the urinary system with a febrile state above 38.5 °C, dysuria, pathological urine sample results along with laboratory markers of inflammation present),
- no antibiotic consumption 3–4 weeks prior to admission to hospital,
- absence of known urologic anomalies,
- absence of circumcision,
- absence of allergy to cow’s milk or any other chronic disease, e.g., otherwise a healthy child.

A detailed interview with the parents and a clinical examination of the child was performed. Complete urine sample testing was performed at all points in the protocol with a complete urine analysis by using dipstick qualitative urine analysis on a Roche Cobas u601 analyser, determining pH, proteins, ketones, glucose, hemoglobin, leukocyte esterase, nitrites, and proteins. Additionally, the numerical counts of erythrocytes, leukocytes, and bacteria in its sediment were determined. A urinary infection was considered when there were leukocytes either 2+ or >10–20/hpf in sediment with bacteria present and urine culture positive with any known uropathogen. In pre-toiled trained children, a clean catch was done. With justified suspicion of a UTI, antibiotic therapy was initiated, and the UTI was confirmed by a positive urine culture with CFU ≥ 10^5^/mL. The treatment of the UTI was planned in line with national recommendations [3]. After the first dose of intravenous antibiotics, children started taking supplements (probiotic/placebo) every day, as one teaspoon of powder for 6 months. Supplements with probiotics and placebos were packed into 50 g plastic bottles at International Probiotic Company s.r.o., Košice, Slovakia, and were marked red and yellow, respectively, but we did not know which bottle contained probiotics and which placebo. Randomization was done by changing the color of the bottle for each subsequent patient included in the study. The white powder consisted of 70% glucose, 15% powdered milk, and 15% whey; the red ones, as we only discovered at the end of the study, consisted of 1 × 10^9^ CFU of Lp26 per teaspoon of powder. When their symptoms abated, they were released to home care with oral antibiotic therapy. Basic blood and urine parameters were determined using standard laboratory techniques. Interleukins (IL) in blood and urine were determined using the Enzyme Linked Immunosorbent Assay (ELISA) tests according to the manufacturer’s recommendations. The aim was to check if the change in IL levels is higher or lower in the test group when compared to the control group. For that reason, we compared the difference between the value of IL level at admission and at discharge in the test (probiotic) group and in the control group. Next, we wanted to check if the IL levels change significantly between discharge and follow-up, especially if there would be cases of reinfection.

Ultrasound evaluation (Alpha 7 Aloka, 2011) of the urinary tract was made consisting of a description of the bladder and kidneys (measurements of kidney length, structure, perfusion, focal signs of inflammation, parenchymal width, urothelial echogenicity, and congenital anomalies). The same protocol was applied after six months.

The nonparametric Mann-Whitney U test and Fisher exact test were used to compare the case and control group. Frieman’s two-way ANOVA for related samples and Wilcoxon Signed Rank Test with Bonferroni correction were used to compare the measurements at admission, upon discharge, and after six months. The numerical measurements are described with a median and 95% confidence interval. Nominal variables are presented with frequencies and proportions. *p*-value less than 0.05 was considered statistically significant.

## 3. Results

Thirty children aged from 2 months to 17 years were included in the study. The median age was 20 months (13, 62). Fourteen children were included in the test group and sixteen in the control group. The age was not significantly different between the groups (U = 85.000; *p* = 0.261). Twenty-four (80%) children included in the study were female. No significant difference in gender distribution between the test and control group was found (*p* = 0.657, Fisher exact test).

Demographic, clinical, ultrasound, blood, and urine parameters measured upon admission are presented in Table 1.

We included only those urine parameters that are important in UTI diagnoses, such as a high number of leukocytes and bacteria in the urine with the presence of nitrites and ketones.

**Figure 2:**
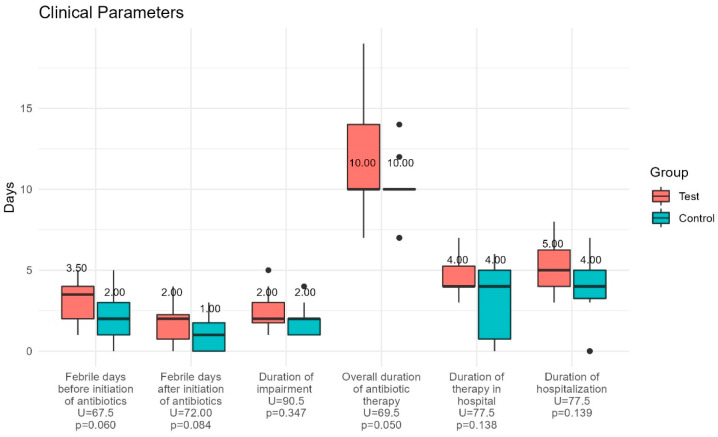
Clinical parameters for the test (probiotic) and control (placebo) group. The duration of febrile days before and after initiation of antibiotic treatment, the overall duration of antibiotic therapy, and hospitalization days showed no statistically significant differences between the groups.

The values of inflammatory markers, e.g., sedimentation rate (SR), C-reactive protein (CRP), procalcitonin (PCT), and leukocytes (WBC), were elevated in accordance with their signs of febrile UTI in all children included. At the admission, most of the children in both groups had high values of WBC and bacteria in their urine. The amounts of nitrites and ketones in urine samples were higher in the test group (nitrites: 50% vs. 18.8%; ketones 21.4% vs. 12.5%); however, the differences were not significant (Table 1). Important clinical parameters are presented in Figure 2.

The duration of febrile days before and after initiation of antibiotic treatment, the overall duration of antibiotic therapy, and hospitalization days showed no statistically significant differences between the groups, although the values were lower in the control group. Specifically, we would point out that children in the control group had fewer febrile days after antibiotic administration compared to the test group (*p* = 0.084). The median values of interleukins in blood and urine at each time point are presented in the Appendix A.

We calculated the differences upon admission and discharge measurements of the basic blood and urine parameters and interleukin levels for each child to check the short-term response to the treatment. Similarly, we calculated the difference between values at discharge and after six months to see if there are some long-term effects of taking probiotics (Table 2). Wilcoxon Signed Rank test for dependent samples showed a statistically significant lowering of all inflammatory parameters in the blood (SR: ptest = 0.009; pcontrol = 0.012, CRP: ptest = 0.009; pcontrol < 0.001, PCT: ptest = 0.015; pcontrol = 0.009, WBC: ptest = 0.003; pcontrol = 0.003) at discharge in comparison with those at admission. Other parameters in blood and urine did not show any statistically significant differences during UTI. When we compared differences among values at discharge and after 6 months there were also no significant differences in those parameters.

Median values of the differences in all results at admission and upon discharge and between discharge and after 6 months in the test and control group were compared. We noticed statistically significant differences in WBC count at the end of the study (*p* = 0.039). The median difference for the test group is −0.5 (−1.40, 0.20) which shows the elevation of WBC count after 6 months. In the control group, the median difference in WBC count was 1.15 (−0.60, 2.10), indicating a lowering of the WBC counts (Table 3). Bacterial load in urine was reduced in 84% of children from the test group and in all children from the control group at discharge. Leukocytes in urine were reduced in all case subjects and 85.7% of control subjects at discharge. The bacteria count in urine was not increased in 63.6% of children from the test group and 84.6% of children in the control group six months after discharge. WBC in urine was not increased in 90.9% of children from the test group and 92.3% of children from the control group six months after discharge. However, the differences were not statistically significant (Table 2).

Next, we compared interleukin levels presented in Table 3.

IL-6 levels in blood samples were significantly reduced at the discharge in the test and the control group (ptest = 0.003, pcontrol = 0.001) compared to admission.

IL-8 levels in blood samples were significantly reduced only in the control group (pcontrol = 0.012). IL-6 and IL-8 were both significantly reduced at discharge compared to admission in the urine sample (ptest = 0.024, pcontrol = 0.039; ptest = 0.003, pcontrol = 0.003). There were no significant differences found in IL levels after 6 months between research and control group except in IL-10 which was significantly higher in the control group when measured in the urine sample (pcontrol = 0.033). Two (12.5%) children in the control group suffered a recurrence of UTI within 6 months after discharge while there was no recurrence of UTI in the test group. Nine (60%) children in the control group and five (41.67%) children in the test group had at least one viral infection within 6 months of discharge. Results of the US evaluation of the urinary tract did not exhibit any statistically significant differences between groups as regards congenital anomalies and signs of inflammation. Testing for probiotics in urine with molecular (PCR) diagnostics after 6 months proved negative. None of the hemocultures were positive. Differences in IgA, G, and M values in blood samples were not significant between our groups. We did not observe any side effects related to the use of probiotics.

## 4. Discussion

In our pilot clinical study, the impact of probiotic strain *Lp26* was investigated in the treatment and prevention of UTIs in children. Our results showed that children with UTIs, who received probiotics alongside classic antibiotic therapy, could suffer a milder course with shortened febrile days and could be at a decreased risk for reinfection; however, the results were not statistically significant.

This is in accordance with the conclusions from a systematic review and meta-analysis of the efficacy of probiotics in UTIs [22]. Other results between the test and the control group were also non-significant, but did show higher values of WBC concentrations after 6 months of follow-up in the control group; however, they were still in the normal reference range for the specific age of children. Although we measured many pro- and anti-inflammatory cytokines in the blood and urine of children in this study, no significant differences between both groups appeared, including interleukin IL-6, an important mediator of the inflammatory response. IL-6 appears to have dual roles in being both protective and deleterious, likely related to at times being anti-inflammatory and at other times being pro-inflammatory [23]. These effects could arise from the systemic influence of probiotics as immunomodulators. Some researchers claim that the downregulation of pro-inflammatory cytokines (IL-2, IL-6, IL-8, TNF-alpha) is the basic mechanism of probiotic function in the treatment of UTI [5,17].

Duell et al. showed that patients with UPEC cystitis secreted significant urinary IL-10 and produced high levels of IL-10 systemically during UPEC urosepsis [24]. Considering the role of IL-10 as a master regulator of innate immunity, which works at the crossroads of inflammation and immunosuppression, we suspected elevated values of IL-10 in urine and blood after consumption of the probiotic strain. However, our results did not confirm that. The function of IL-10 in the infected human urinary tract is still unknown [24]. The complicated nature of individuals’ immune response, which is also seen in the dynamics of the cytokine response that we observed in blood and urine, was consistent with the results in the literature [5,6,25,26,27,28,29]. As Hussein et al. emphasized in their work, we are not sure at present whether elevated urinary interleukin levels reflect bacterial virulence or the host’s ability to clear the infection. In addition, it has been shown that elevated IL-8 in tissue is essential for the treatment of bacterial infection, however, it can also lead to tissue damage [6].

It has also been suggested that probiotic bacteria that promote health through driving mucosal immune mechanisms, compared to those with strictly local effects, such as ‘immunobiotics’, for immunomodulation at distant mucosal sites have to be found [25,26]. In our pilot study testing for probiotics in urine with molecular (PCR) diagnostics after 6 months proved negative. Aiming to reduce uropathogens by replacing them with probiotics, their absence in the urine points to the fact that we were not able to access colonization of *Lp26* in the periurethral region. Such a fact does not substantiate the assumption that probiotics can travel from the gut all the way to the periurethral flora; however, studies of adult women did demonstrate this mechanism of colonization of the vagina [18]. Possibly, a different regime (twice a day) and higher dosage of probiotic strains would be more appropriate to achieve this effect and replace other e.g., uropathogenic bacteria [12]. Additionally, a larger sample size may have yielded statistically significant results.

One of the goals of the study was to determine the effectiveness of probiotics in UTI prophylaxis. Our results showed that two children in the control group suffered a recurrence of febrile UTI within 6 months of the follow-up period, while there were no recurrences of UTI in the test group. We realize that small study groups preclude us from definite conclusions, however, we consider these promising results of probiotic UTI prophylaxis. We have not found any clinically important scars with US examination. No significant differences were observed in the mentioned parameters between the groups. We are of the opinion that the US is a useful diagnostic tool for detecting large renal scars related to chronic kidney disease (CKD). Naturally, it is possible that smaller changes in kidney parenchyma, which are not clinically important, went undetected. We don’t feel using more invasive imaging exam techniques solely for scientific purposes would be particularly ethical. In our research-case, this would involve static kidney scintigraphy or magnetic resonance imaging (MRI) of the urinary tract under general anesthesia, which we do not routinely employ for managing otherwise healthy children with UTIs. The concept of ALARA (“As Low As Reasonably Achievable”) and the *Image Gently* campaign, to which pediatric radiologists commonly subscribe, teaches exactly this.

The greatest limitation of our study is the small sample size. It was challenging to recruit patients to adhere to the protocol and some of them had to be excluded due to non-compliance. Consequently, the large age range presents another limitation, since the pathophysiology differs greatly between the youngest and the oldest. Additionally, a specific strain used showed in vitro effect on *E.coli*, but was not tested on other uropathogens that were also present in our cohort. Given all the limitations, it is really surprising that the research showed indicative results of probiotic benefits in UTIs in this small and diverse cohort, emphasizing the need for further and expanded research.

## 5. Conclusions

Our clinical pilot study investigated the potential impact of probiotics on the treatment and prevention of UTI in otherwise healthy children and it achieved some promising results. Namely, children on antibiotic therapy for UTI alongside probiotic supplementation suffered milder clinical courses of infection with shorter duration of febrile state compared to children on the same classic antibiotic treatment regime with placebo during UTI according to some laboratory parameters, but the results were statistically non-significant. The following check-ups carried out on children with probiotic supplementation also provided favorable results, as there were no recurrences of UTI in the 6-month period. Anyway, both the effects on the course and the recurrence did not yield noticeable statistical significance. As the sample size of our pilot study was small and the groups of children heterogeneous, larger prospective trials would be needed to draw firm conclusions on the matter. We also stress that better basic scientific studies for the selection of proper immunobiotic strains of probiotics for the treatment and prevention of UTI and the regime of consumption should be performed to aid researchers in selecting the most appropriate probiotic strain.

## Figures and Tables

**Table 1:** Demographic, clinical, laboratory, and ultrasound parameters measured upon admission in the test and control group.

			Test (N = 14)	Controls (N = 16)	
			N (%)	N (%)	χ^2^ (*p*)
Gender	[female]		12 (85.7%)	12 (75.0%)	(0.657) *
Body temperature at admission	[over 39 °C]		4 (28.6%)	5 (31.3%)	(1.000) *
Irritability	[yes]		5 (35.7%)	7 (43.8%)	0.201 (0.654)
Food refusal	[yes]		4 (28.6%)	4 (25.0)	(1.000) *
*E. coli*	[positive]		11 (78.6%)	15 (93.8%)	(0.315) *
Constipation	[yes]		3 (21.4%)	1 (6.3%)	(0.315) *
			**Median (95% CI)**	**Median (95% CI)**	**U (*p*)**
Age	[months]		51.5 (13, 120)	52 (10, 90)	103.500 (0.724)
Blood parameters	SR [mm/h]		40 (14, 73)	63.5 (48, 76)	66.500 (0.059)
	CRP [mg/dL]		56.9 (17.9, 118.6)	92.6 (85.9, 129.0)	71.000 (0.088)
	PCT [μg/L]		0.30 (0.10, 2.80)	0.95 (0.30, 4.20)	72.000 (0.096)
	GLU [mmol/L]		5.42 (4.90, 5.85)	4.89 (4.43, 5.10)	62.500 (0.103)
	Leukocytes [×10^9^/L]		15.95 (10.40, 19.70)	19.05 (16.00, 23.30)	84.000 (0.244)
	Creatinine [umol/L]		36 (28, 54)	36 (31, 46)	98.000 (0.760)
	Urea [mg/L]		3.6 (2.6, 4.1)	3.7 (3.2, 5.2)	76.500 (0.213)
	PMN [%]		59.4 (51.0, 74.5)	62.0 (59.2, 72.2)	91.000 (0.541)
	Hb [g/L]		117 (103, 128)	110 (102, 116)	77.500 (0.229)
Urine parameters	pH urine		6.00	6.00 (6.00, 7.00)	98.000 (0.509)
		**Value**	**N (%)**	**N (%)**	**χ^2^ (*p*)**
	Leukocytes	high	11 (78.6%)	14 (87.5%)	(0.642) *
	Bacteria	high	12 (85.7%)	13 (81.3%)	(1.000) *
	Nitrites	yes	7 (50.0%)	3 (18.8%)	(0.122) *
	Ketones	yes	3 (21.4%)	2 (12.5%)	(0.642) *
Ultrasound parameters	Thickening of the urothelium	yes	8 (57.1%)	5 (31.3%)	2.039 (0.153)
	Enlarged kidneys	one/both	8 (57.1%)	6 (40.0%)	0.852 (0.356)
	No signs of upper urinary tract inflammation	positive	4 (28.6%)	1 (6.3%)	(0.157) *

* Fisher exact test *p* value.

**Table 2:** Comparison of the basic blood and urine parameters measured at admission, on discharge and after six months.

			Test		Control		
			Median Difference (95% CI)	χ^2^ (*p*) °	Median Difference (95% CI)	χ^2^ (*p*) °	U (*p*) *
Admission—Discharge	Blood	SR [mm/h]	20 (5, 42)	**6.00 (0.009)**	35.5 (20, 57)	**6.00 (0.012)**	83.00 (0.490)
		CRP [mg/L]	65.1 (37.9, 117.2)	**0.00 (0.003)**	86.1 (80.1, 128.4)	**0.00 ( <0.003)**	66 (0.096)
		PCT [μg/L]	0.43 (0.10, 2.86)	**0.00 (0.015)**	0.89 (0.3, 4.18)	**0.00 (0.009)**	35.5 (0.170)
		GLU [mmol/L]	0.41 (−0.50, 0.98)	22.50 (1.000)	0.35 (−0.77, 0.72)	28.00 (1.000)	53.5 (0.640)
		WBC [×10^9^/L]	4.65 (2.70, 11.60)	**1.00 (0.003)**	8.4 (5.8, 12.4)	**0.00 (0.003)**	69 (0.116)
		creatinine [umol/L]	0.5 (−1.0, 50.0)	31.00 (1.000)	1.5 (−2, 1)	33.00 (1.000)	90 (0.712)
		PMN [%]	19.8 (13.1, 28.3)	**0.00 (0.006)**	26.8 (18, 28.8)	**0.00 (0.003)**	64 (0.190)
		Hb [g/L]	0.5 (−7.0, 3.0)	49.00 (1.000)	−0.5 (−16, 1)	49.00 (0.462)	81 (0.433)
	Urine	pH	0.0 (−1.0, 1.0)	36.00 (1.000)	−0.7 (−1, 0)	48.00 (0.096)	69 (0.273)
			**Value**	**n/N (%)**	**n/N (%)**		**Fisher (*p*)** **^x^**
		Bacteria	reduced ^	11/13 (84.6%)	14/14 (100%)		NA
		Leukocytes	reduced ^	13/13 (84.6%)	12/14 (85.7%)		NA
Discharge—After 6 months	Blood	SR [mm/h]	9.5 (1.0, 18.0)	5.00 (0.735)	9.0 (−1, 26)	3.00 (0.189)	20 (0.886)
		CRP [mg/L]	0.1 (−3.2, 6.0)	14.00 (1.000)	−0.2 (−8.1, 3.4)	30.00 (1.000)	33.5 (0.883)
		WBC [*×*10^9^*/*L]	−0.5 (−1.40, 0.20)	49.00 (0.462)	1.15 (−0.60, 2.10)	15.50 (0.192)	**32.5 (0.039)**
		CREATININE [umol/L]	2.0 (−3.0, 7.0)	28.50 (1.000)	1.0 (−4.0, 70)	45.00 (1.000)	62.5 (0.601)
		PMN [%]	−3.2 (−10.5, 8.7)	33.00 (1.000)	3.4 (−5.4, 14.9)	13.00 (0.780)	32 (0.288)
		Hb [g/L]	−6.0 (−7.0, 7.0)	47.50 (0.588)	−6.5 (−11.0, −3.0)	**66.00 (0.009)**	54 (0.459)
	Urine	pH	0.00 (−1.00, 1.00)	23.00 (1.000)	−0.25 (−1.00, 1.00)	32.00 (1.000)	63 (0.851)
			**Value**	**n/N (%)**	**n/N (%)**		**Fisher (*p*)** **^x^**
		Bacteria	not increased ˇ	7/11 (63.6%)	11/13 (84.6%)		(0.357)
		Leukocytes	not increased ˇ	10/11 (90.9%)	12/13 (92.3%)		(1.000)

* Mann Whitney U test on the difference between IL levels at (1) admission and discharge and (2) discharge, and after 6 months. ° Wilcoxon signed rank test for related samples on measured IL levels at (1) admission and discharge and (2) discharge and after 6 months; significance adjusted with Bonferroni correction. ^x^ Fisher exact test. ^ significant reduction to near normal levels in comparison to laboratory value at admission indicating an appropriate response to the treatment. ˇ no significant increment in comparison to the laboratory value at discharge that would indicate reinfection. Significance level *p* < 0.05 marked bold.

**Table 3:** Comparison of the interleukin levels in blood and urine measured at admission, on discharge, and after six months.

			Test		Control		
			Median Difference (95% CI)	χ^2^ (*p*) °	Median Difference (95% CI)	χ^2^ (*p*) °	U (*p*) *
Admission—Discharge	Blood	IL2	0.12 (−0.25, 2.22)	36.00 (0.900)	0.35 (−0.12, 0.83)	62.00 (1.000)	97.00 (0.533)
		IL6	8.37 (3.82, 17.97)	1.00 (0.003)	12.44 (5.43, 28.71)	**0.00 (0.001)**	87.00 (0.299)
		IL8	6.08 (−0.36, 30.41)	20.00 (0.123)	4.63 (2.35, 11.73)	**12.00 (0.012)**	111.00 (0.967)
		IL10	3.74 (1.22, 19.52)	15.00 (0.057)	5.48 (1.31, 15.45)	29.00 (0.132)	108.00 (0.868)
		IL12	0.04 (−0.61, 0.28)	52.00 (1.000)	−0.17 (−1.1, 0.3)	73.00 (1.000)	98.00 (0.561)
		TNFa	0.09 (−1.62, 1.19)	46.00 (1.000)	0.05 (−0.91, 1.53)	66.00(1.000)	107.00 (0.585)
	Urine	IL6	10.87 (0.57, 91.26)	10.00 (0.024)	8.49 (0.11, 14.79)	13.00 (0.039)	81.00 (0.435)
		IL8	241.95 (29.66, 624.95)	1.00 (0.006)	128.55 (64.19, 193.66)	0.00 (0.003)	86.00 (0.581)
		IL10	0.13 (−0.01, 32.94)	17.00 (0.078)	0.09 (−0.13, 0.24)	37.00 (0.993)	71.00 (0.215)
		IL12	−0.16 (−0.52, 0.08)	62.003 (0.747)	0.00 (−0.13, 0.33)	34.00 (1.000)	79.50 (0.395)
Discharge—After 6 months	Blood	IL2	0.35 (−0.12, 0.83)	15.00 (0.180)	0.28 (−0.02, 1.47)	27.00 (0.588)	72.00 (0.744)
		IL6	0.00 (−1.23, 0.55)	42.00(1.000)	0.49 (−1.73, 1.22)	45.00 (1.000)	69.00 (0.44)
		IL8	1.12 (−3.72, 6.71)	30.00 (1.000)	−2.98 (−5.84, 4.94)	35.00 (1.000)	61.00 (0.237)
		IL10	0.96 (−0.13, 2.19)	18.00 (0.297)	1.96 (−5.24, 5.58)	41.00 (1.000)	77.00 (0.719)
		IL12	0.36 (−1.75, 1.82)	37.00 (1.000)	−0.56 (−2.23, 1.13)	69.00 (0.900)	67.00 (0.382)
		TNFa	0.27 (−2.40, 5.70)	29.00 (1.000)	0.33 (−2.25, 2.74)	37.00 (1.000)	83.50 (0.979)
	Urine	IL6	−0.01 (−0.9, 1.56)	31.00 (1.000)	0.37 (−0.71, 3.15)	30.00 (0.837)	63.00 (0.415)
		IL8	−0.39 (−17, 5.74)	44.00 (1.000)	−0.19 (−2.2, 38.46)	39.00 (0.474)	69.00 (0.624)
		IL10	−0.12 (−0.48, 0.45)	92.00 (1.000))	−0.36 (−0.55, 0.09)	82.00 (0.033)	49.00 (0.115)
		IL12	−0.28 (−0.82, 0.41)	50.00 (1.000)	−0.32 (−0.78, 0.41)	67.00 (0.399)	70.00 (0.663)

* Mann Whitney U test on the difference between IL levels at (1) admission and discharge and (2) discharge, and after 6 months. ° Wilcoxon signed rank test for related samples on measured IL levels at (1) admission and discharge and (2) discharge and after 6 months; significance adjusted with Bonferroni correction. Significance level *p* < 0.05 marked bold.

## Data Availability

All the data is available at the corresponding author upon reasonable request.

## Appendix A

**Table A1:** Interleukins measured in blood and urine samples upon admission to the hospital, discharge, and after 6 months in the case and the control group (all values in pg/mL).

			Test	Control	
			Median (95% CI)	Median (95% CI)	U (*p*)
Admission	Blood	IL2	1.03 (0.44; 2.22)	0.68 (0.22; 1.22)	94.00 (0.454)
		IL6	19.09 (5.93; 19.32)	14.05 (10.98; 31.08)	105.00 (0.360)
		IL8	18.83 (8.69; 39.39)	17.71 (15.01; 21.85)	110.50 (0.647)
		IL10	13.42 (7.3; 24.1)	19.66 (10.48; 26.31)	92.50 (0.618)
		IL12	2.63 (0.35; 5.44)	1.66 (0.59; 2.92)	101.00 (0.589)
		TNFα	9.15 (1.66, 12.06)	3.83 (1.40, 6.18)	81.50 (0.205)
	Urine	IL6	11.80 (0.57; 92.06)	10.60 (1.83; 18.13)	57.00 (0.934)
		IL8	374.03 (68.48; 660.23)	131.14 (90.76; 242.14)	94.00 (0.280)
		IL10	0.25 (0.10; 33.44)	0.22 (0.07; 0.31)	85.00 (0.236)
		IL12	0.54 (0.32; 0.80)	0.37 (0.05; 0.94)	67.00 (0.298)
Discharge	Blood	IL2	0.70 (0.44; 1.17)	0.51 (0.16; 1.53)	104.50 (0.755)
		IL6	1.70 (1.38; 2.81)	1.65 (1.21; 3.00)	105.00 (0.771)
		IL8	9.25 (8.82; 13.05)	9.65 (8.51; 14.87)	110.50 (0.950)
		IL10	6.55 (4.39; 12.75)	8.36 (5.13; 13.61)	92.50 (0.418)
		IL12	2.73 (1.55; 3.68)	1.99 (0.76; 3.56)	101.00 (0.647)
		TNFα	9.57 (3.02, 11.88)	3.57 (1.38, 12.76)	95.50 (0.493)
	Urine	IL6	0.30 (0.27; 1.10)	1.14 (0.49; 3.15)	57.00 (0.060)
		IL8	3.22 (0.86; 13.59)	3.96 (0.59; 15.11)	94.00 (0.854)
		IL10	0.17 (0.05; 0.64)	0.17 (0.02; 0.51)	85.00 (0.550)
		IL12	0.65 (0.38; 1.41)	0.23 (0.03; 1.35)	67.00 (0.154)
After six months	Blood	IL6	0.49 (0.02; 0.98)	0.17 (0.06; 3.11)	74.00 (0.828)
		IL6	1.92 (1.03; 2.87)	1.26 (0.29; 6.14)	82.00 (0.918)
		IL8	9.27 (4.42; 18.24)	13.73 (10.08; 25.68)	46.00 (0.051)
		IL10	5.00 (2.10; 11.39)	9.14 (5.89; 11.10)	56.00 (0.150)
		IL12	3.02 (0.77; 7.89)	1.88 (1.03; 11.16)	75.00 (0.643)
		TNFα	7.24 (2.83, 15.51)	3.76 (0.89, 6.92)	60.50 (0.227)
	Urine	IL6	0.47 (0.00; 1.22)	0.11 (0.00; 1.21)	78.50 (0.766)
		IL8	11.92 (1.92; 23.02)	6.14 (2.52; 17.01)	68.00 (0.411)
		IL10	0.44 (0.24; 0.62)	0.53 (0.22; 0.65)	74.00 (0.607)
		IL12	0.94 (0.50; 1.50)	0.83 (0.45; 0.94)	69.00 (0.440)
